# Supplementary material for: Identification of photoperiod-induced specific miRNAs in the adrenal glands of Sunite sheep (Ovis aries)
Source: Front Vet Sci. 2022 Jul 22;9:888207. doi: 10.3389/fvets.2022.888207 (PMC9354845; doi:10.3389/fvets.2022.888207)
Supplement: Supplementary file 1 [file Data_Sheet_1.ZIP › Supplementary file/miRDeep2 file.zip!/miRDeep2 file/Know_Structure/SPLP42A2/oar-mir-362.pdf]

miRBase precursor	: oar-mir-362
Total read count	: 1248
oar-miR-362 read count	: 1229
remaining reads	: 19

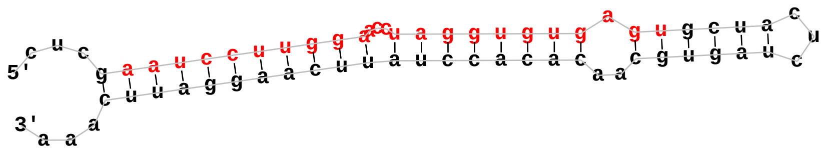

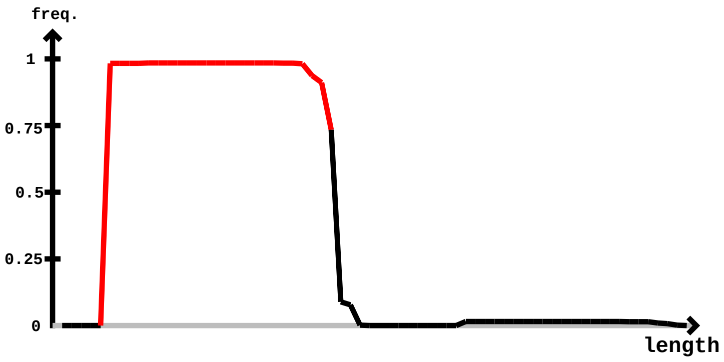


```
      oar-miR-362
5'- cucgaauccuuggaaccuaggugugagugcuacucuagugcaacacaccuauucaaggauucaaa   -3'    exp
    ...(((((((((((...((((((((.((((((...))))))..)))))))))))))))))))...    reads      mm         sample
    ....aauccuuggaaccuaggu...........................................    1          0          seq
    ....aauccuuggaaccuaggugu.........................................    3          0          seq
    ....aauccuuggaaccuaggugug........................................    55         0          seq
    ....aauccuuggaaccuagguguga.......................................    33         0          seq
    ....aauccuuggaaccuaggugugag......................................    221        0          seq
    ....aauccuuggaaccuaggugugagu.....................................    804        0          seq
    ....aauccuuggaaccuaggugugagug....................................    14         0          seq
    ....aauccuuggaaccuaggugugagugc...................................    94         0          seq
    ....aauccuuggaaccuaggugugagugcu..................................    2          0          seq
    ........cuuggaaccuaggugugagu.....................................    1          0          seq
    ........cuuggaaccuaggugugagugc...................................    1          0          seq
    .........................................aacacaccuauucaagg.......    1          0          seq
    .........................................aacacaccuauucaaggauu....    6          0          seq
    .........................................aacacaccuauucaaggauuc...    3          0          seq
    .........................................aacacaccuauucaaggauuca..    7          0          seq
    .........................................aacacaccuauucaaggauucaa.    2          0          seq
```
